# Supplementary material for: A Pilot Study on Patient-specific Computational Forecasting of Prostate Cancer Growth during Active Surveillance Using an Imaging-informed Biomechanistic Model
Source: Cancer Res Commun. 2024 Mar 1;4(3):617–33. doi: 10.1158/2767-9764.CRC-23-0449 (PMC10906139; doi:10.1158/2767-9764.CRC-23-0449)
Supplement: Supplementary Methods [file crc-23-0449-s01.docx]

| **Supplementary Methods** |
| --- |

**A pilot study on patient-specific computational forecasting of prostate cancer growth during active surveillance using an imaging-informed biomechanistic model**

Guillermo Lorenzo^1,2^, Jon S. Heiselman^3,4^, Michael A. Liss^5^, Michael I. Miga^3,6,7^, Hector Gomez^8^, Thomas E. Yankeelov^2,9,10^, Alessandro Reali^1^, Thomas J. R. Hughes^2^

^1^Department of Civil Engineering and Architecture, University of Pavia, Italy

^2^Oden Institute for Computational Engineering and Sciences, The University of Texas at Austin, USA

^3^Department of Biomedical Engineering, Vanderbilt University, USA

^4^Department of Surgery, Memorial Sloan-Kettering Cancer Center, USA

^5^Department of Urology, University of Texas Health San Antonio, USA

^6^Vanderbilt Institute for Surgery and Engineering, Vanderbilt University, USA

^7^Department of Neurological Surgery, Radiology, and Otolaryngology-Head and Neck Surgery, Vanderbilt University Medical Center, USA

^8^School of Mechanical Engineering, Weldon School of Biomedical Engineering, and Purdue Institute for Cancer Research, Purdue University, USA

^9^Livestrong Cancer Institutes and Departments of Biomedical Engineering, Diagnostic Medicine, and Oncology, The University of Texas at Austin, USA

^10^Department of Imaging Physics, The University of Texas MD Anderson Cancer Center, USA

**Corresponding author:**

Guillermo Lorenzo, PhD.

Department of Civil Engineering and Architecture

University of Pavia,

Via Ferrata 3, 27100, Pavia, Italia.

Email: [guillermo.lorenzo@unipv.it](mailto:guillermo.lorenzo@unipv.it), [guillermo.lorenzo@utexas.edu](mailto:guillermo.lorenzo@utexas.edu)

**Supplementary Methods**

**S1. Hyperbolic tangent model fit for ADC ratio and continuous Gleason score**

The apparent diffusion coefficient (ADC) measured *via* diffusion-weighted magnetic resonance imaging (DW-MRI) is known to decrease in prostate cancer (PCa) cases exhibiting higher Gleason score (GS), as reported in Refs. (41-45) in the main text. We exploited this observation for the semi-automatic segmentation of prostatic tumors after the manual delineation of a gross region of interest of the tumor lesion (see subsection on preprocessing of the imaging data in the Materials and Methods). Toward this end, we defined a quantitative relationship between ADC and GS, such that we could automatically identify the subregion of the gross tumor delineation with values of ADC representing mpMRI-observable tumors, which usually exhibit GS ≥ 3+3. To facilitate the application of our methods to ADC maps obtained for each patient at different time points and across patients, we leveraged the ADC ratio ($ADC_{r}=ADC/ADC_{h}$, where $ADC_{h}$ is a representative measurement of ADC in each patient’s healthy prostatic tissue), which has also been shown to exhibit a decreasing trend with increasing Gleason score (41-45). Furthermore, we extended the discrete definition of GS used in the clinic, whereby this metric can only take natural numbers in the range [2, 10], to a continuous definition in the range [0, 10]. Hence, the values in the interval [0, 2] are assumed to represent healthy and pre-tumoral lesions. The rationale for the continuous definition was to account for the changing ADC as the tumor evolves toward higher GS, such that these continuous dynamics could then be matched to the spatiotemporal evolution of the normalized tumor cell density maps calculated with our biomechanistic model of PCa growth. To capture the decrease in the ADC ratio with increasing GS, we adopted a hyperbolic tangent function because it provided two horizontal asymptotes that can be used to account for two additional features of interest: (i) a plateauing trend in the decrease of ADC ratio for the highest GS values, which aligns with a higher tumor cell density approaching tissue carrying capacity in those tumors (see Refs. (46-48) in the main text); and (ii) a plateau for values of ADC ratio close to zero (i.e., healthy tissue) followed by a slow decrease of the ADC ratio in pre-tumoral lesions and low GS tumors, which makes them practically indistinguishable from healthy tissue and matches the inability of mpMRI to accurately identify PCa cases with GS < 3+3.

The mathematical formulation of the hyperbolic tangent function that we used in this work is given by

| $ADC_{r}=a-b\tanh(c(GS-d)),$ | (S1) |
| --- | --- |

where $a$, $b$, $c$, and $d$ are empirical fitting constants. We enforced $ADC_{r}$= 1 for $GS=$ 0, such that the ADC in healthy tissue satisfied $ADC=ADC_{h}$. By introducing this constraint in Eq. (S1), we could eliminate parameter $a$ and simplify the fitting problem to the determination of the other empirical constants using the following equation:

| $ADC_{r}=1+b\left( \tanh\left( -cd \right)-\tanh\left( c\left( GS-d \right) \right) \right).$ | (S2) |
| --- | --- |

We calculated the values of the empirical constants $b$, $c$, and $d$ that provided the best fit of Eq. (S2) to the average of the ADC ratios obtained from the mean $ADC_{h}$ and mean GS-specific ADC values reported in the five studies mentioned above (i.e., Refs. (41-45) in the main text). Curve fitting was carried out using the *fit* function from the Curve Fitting Toolbox in MATLAB (R2021b; The Mathworks, Natick, MA). The maximum number of iterations and model evaluations were both set at 10,000. The admissible range of values for the empirical constants were $b\in$ [0, 1], $c\in$ [0.30, 0.50], and $d\in$ [5, 7]. The initial guess of these empirical constants was set at the middle point of their corresponding admissible value range. Hence, curve fitting resulted in the following definition of Eq. (S1):

| $ADC_{r}=0.64-0.39\tanh\left( 0.3\left( GS-5 \right) \right).$ | (S3) |
| --- | --- |

The lower asymptote of the hyperbolic tangent function in Eq. (S3) can be calculated as the limit of $ADC_{r}$ when $GS\to\infty$, and was used to define $ADC_{min}\approx$ 0.25$ADC_{h}$. This quantity represents the minimum admissible value of the ADC ratio in PCa, and it enabled the conversion of ADC map measured over a patient’s tumor to a normalized tumor cell density map ($\hat{N}(\boldsymbol{x},t)$) *via* the linear mapping given by

| $\hat{N}\left( \boldsymbol{x},t \right)=\frac{ADC_{h}-ADC(\boldsymbol{x}, t)}{ADC_{h}-ADC_{min}} .$ | (S4) |
| --- | --- |

Importantly, Eq. (S4) accounts for the decrease in ADC with increasing tumor cell density in higher GS tumors (see Refs. (41-48) in the main text). Furthermore, Eq. (S4) has been used in the preprocessing step of the computational pipeline of several successful tumor forecasting studies employing mpMRI-informed biomechanistic models (see Refs. (18, 21, 28, 29) in the main text).

**S2. Interscan registration**

For each patient, the three mpMRI datasets collected during active surveillance (AS) and their corresponding segmentations were co-registered to a common data frame, which was set as that of the first mpMRI scan (see Figure 1C in the main document). This interscan registration was carried out using a biomechanically-constrained, deformable image registration algorithm to control for bulk deformations of the prostate anatomy between scan dates while preserving imaging features associated with longitudinal disease-related changes. The registration process consisted of a rigid registration of the prostate segmentations based on the *T_2_*-weighted MRI (T2W) images followed by a deformable linearized iterative boundary reconstruction algorithm. A robust rigid alignment of prostate anatomy was established by a semiautomatic, salient feature weighted, iterative closest point algorithm (see Ref. (49) in the main text). Salient features were marked from base to apex along the posterior, left, and right aspects of the prostate to aid initial anatomical alignment. The linearized iterative boundary reconstruction algorithm (see Ref. (50) in the main text) was then employed to solve for an optimal distribution of external forces applied to the outer boundary of the prostate to maximize alignment between source and target prostate geometries while preserving biomechanical consistency. Briefly, a series of 90 control points were uniformly distributed over the boundary of the prostate *via* k-means clustering, which partitioned the prostate surface into an equal number of Voronoi control surfaces. Linear elastic biomechanical perturbations of control points to 5-mm displacements in each Cartesian direction were simulated using a finite-element model of the prostate. Near-field displacement responses to these control point perturbations were relaxed over their respective control surfaces according to the Saint-Venant principle by re-equilibrating the local boundary forces within the active control surface against far-field displacements resulting from the point perturbation. These relaxed perturbation responses establish a superposed basis of elastic deformation responses to locally decomposed mechanical loads that are regionally applied over the prostate boundary. A linear combination of this superposed basis subsequently encodes a parameterized deformation response to a continuous, spatially varying distribution of mechanical forces applied over the prostate surface. This parameterized model for whole-organ prostate deformations was then optimized to minimize the distance between the source and the target prostate segmentation boundaries with regularization by a strain energy penalty function to resolve discrepant external forces applied to the prostate across mpMRI scans. Further details are found in Ref. (50) of the main text. This registration method filters out purely elastic deformation effects between imaging dates while preserving the effect of non-elastic physiological changes associated with tumor growth that may occur during AS. As a final step, the interscan registration was also applied to the T2W data, the ADC maps, and the tumor segmentations of each patient. After the interscan registration, we updated the reference prostate segmentation (i.e., that defined in the T2W image of the first mpMRI scan) to prepare it for integration within our modeling framework by subtracting the urethral region. To do this, we used the eraser tool in 3DSlicer with a spherical geometry and 4-mm diameter. Anatomical landmarks in the T2W data were used for reference.
