# Supplementary material for: A Pilot Study on Patient-specific Computational Forecasting of Prostate Cancer Growth during Active Surveillance Using an Imaging-informed Biomechanistic Model
Source: Cancer Res Commun. 2024 Mar 1;4(3):617–33. doi: 10.1158/2767-9764.CRC-23-0449 (PMC10906139; doi:10.1158/2767-9764.CRC-23-0449)
Supplement: Supplementary Figure S2 — Examples of personalized calculations of PCa growth in the fitting-forecasting scenario. [file crc-23-0449-s03.docx]

**
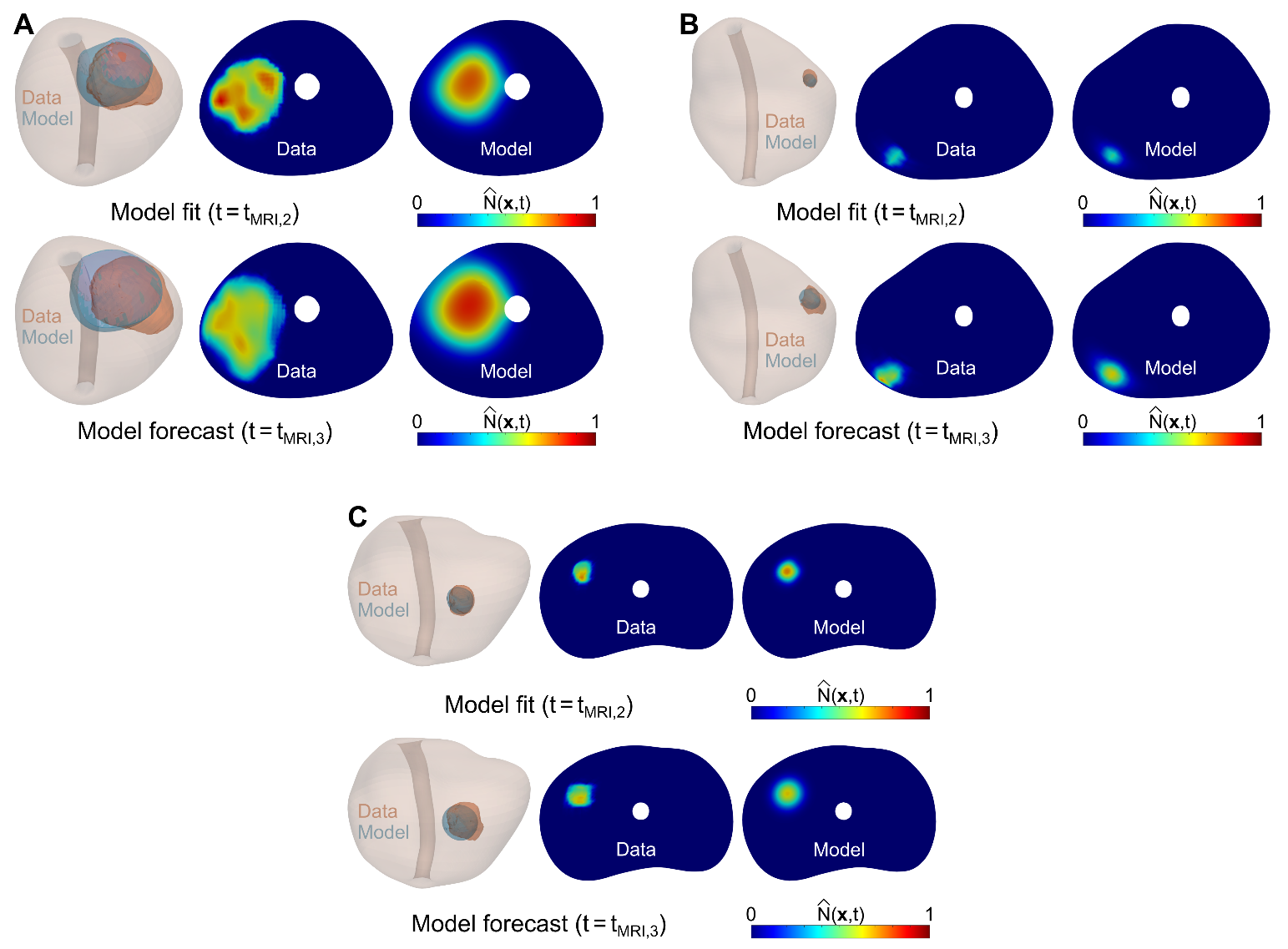
**

**Supplementary Figure S2. Examples of personalized calculations of PCa growth in the fitting-forecasting scenario.** Panels A-C illustrate PCa growth at the date of the 2^nd^ mpMRI scan during model calibration ($t_{MRI,2}$; upper row in each panel) and the ensuing prediction of tumor growth at the date of the 3^rd^ mpMRI scan ($t_{MRI,3}$; bottom row in each panel) for the remaining three patients not shown in Figure 6 of the main text. The patients shown in this figure are the same considered in Supplementary Figure S1. These results are illustrated using a 3D representation of the mpMRI-extracted prostate geometry of each patient including the imaging-measured and model-calculated tumor regions (red and blue volumes), along with an axial section of the prostate showing the normalized tumor cell density map obtained from the mpMRI data and using the personalized model (i.e., Eq. (3) in the main text).
