# Supplementary material for: A Pilot Study on Patient-specific Computational Forecasting of Prostate Cancer Growth during Active Surveillance Using an Imaging-informed Biomechanistic Model
Source: Cancer Res Commun. 2024 Mar 1;4(3):617–33. doi: 10.1158/2767-9764.CRC-23-0449 (PMC10906139; doi:10.1158/2767-9764.CRC-23-0449)
Supplement: Supplementary Figure S3 — Patient-specific forecasts of PCa risk. [file crc-23-0449-s04.docx]

**
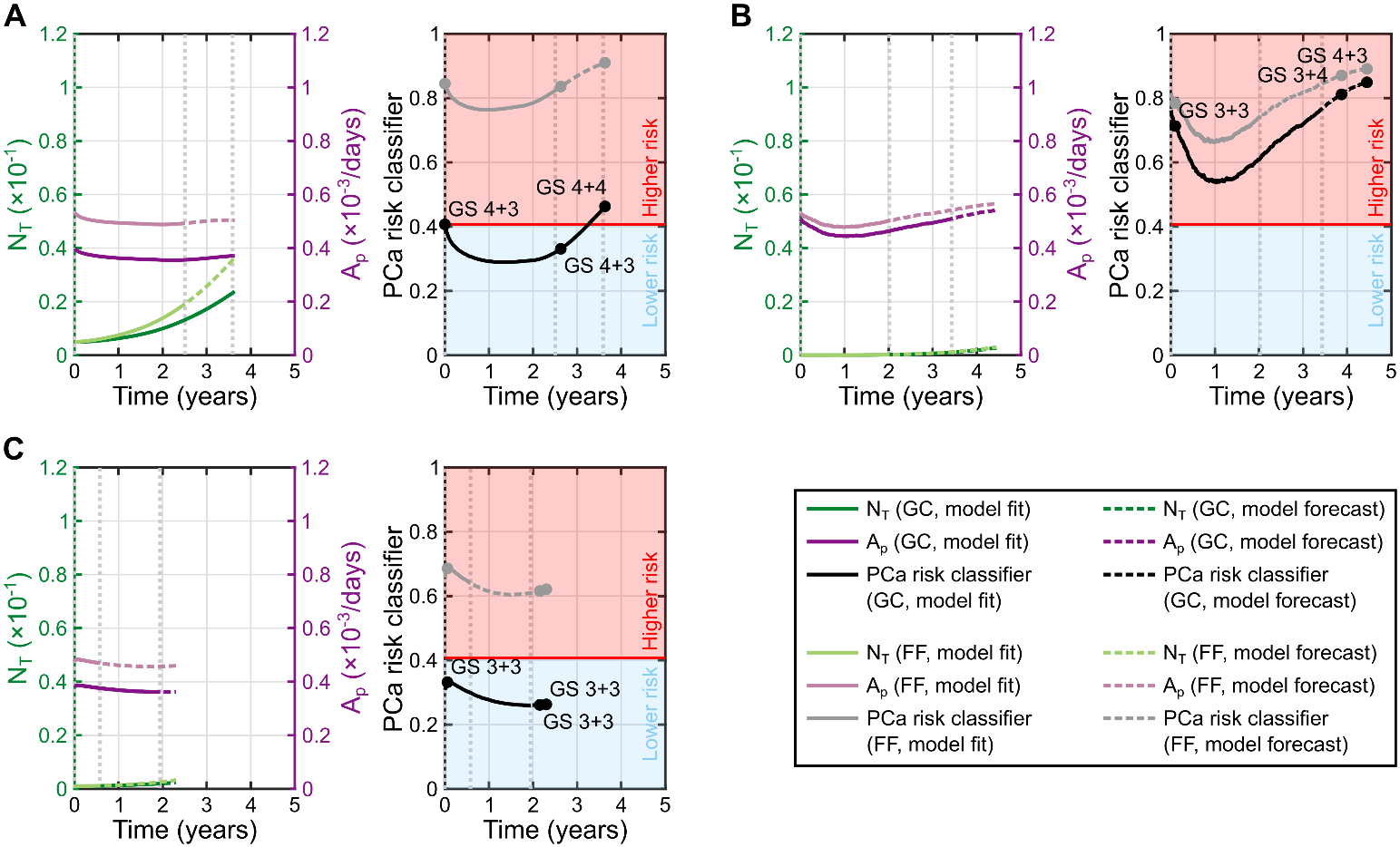
**

**Supplementary Figure S3. Patient-specific forecasts of PCa risk.** Panels A-C show the time trajectories of the model-based markers involved in the calculation of our PCa risk classifier (left) as well as the trajectory of the latter (right) for the remaining three patients not shown in Figure 9 of the main text, respectively. The patients shown in this figure are the same considered in Supplementary Figures S1 and S2. In all panels, darker hues represent results from the global calibration scenario (GC, see Supplementary Figure S1), where the model is fit to the three mpMRI datasets from each patient, while lighter hues show results from the fitting-forecasting scenario (FF, see Supplementary Figure S2), where the model is only fit to the first two mpMRI datasets from each patient. Dotted gray vertical lines in the background indicate the times of the mpMRI scans for each patient. Additionally, solid lines correspond to quantities calculated from the model fit, while dashed lines correspond to values calculated from model forecasts. The model-based markers of interest are the total tumor index ($N_{T}$, green curves, left vertical axis) and mean proliferation activity of the tumor ($A_{p}$, pink curves, right vertical axis). The PCa risk classifier was trained with the global calibration results (see Figure 8 in the main text), yielding an optimal performance threshold that separates lower risk PCa (blue region) from higher-risk PCa (red region). The PCa risk at the times of histopathological assessment of the patients’ tumors (i.e., biopsy, surgery) is represented as a bullet point, and the corresponding GS values are annotated over the PCa risk trajectory from the global calibration scenario. In panel A, in the fitting-forecasting scenario the PCa risk classifier consistently identifies the tumor as a higher-risk case and anticipates tumor progression from GS 4+3 to GS 4+4 using the personalized predictions at the second imaging timepoint, which is 410 days (i.e., ~1.1 years) earlier than the final histopathological assessment at surgery. The global calibration scenario confirms higher-risk disease at the final histopathological assessment, but suggests a lower risk at the times of the first two mpMRI scans and biopsies. In panel B, the tumor is consistently classified as a higher-risk case in both computational scenarios. In this case, the PCa forecasts after the second mpMRI scan in the fitting-forecasting scenario enable the detection of tumor progression 677 days (i.e., ~1.9 years) before surgery. The tumor in panel C is consistently classified as a higher-risk case using the predictions from the fitting-forecasting study, but assimilation of the third mpMRI dataset in the global calibration scenario correctly classified the tumor as a lower-risk case.
